# cMet agonistic antibody attenuates apoptosis in ischaemia‐reperfusion–induced kidney injury

**DOI:** 10.1111/jcmm.15225

**Published:** 2020-04-02

**Authors:** Jung Nam An, Lilin Li, Junghun Lee, Seung‐Shin Yu, Jeonghwan Lee, Yong Chul Kim, Dong Ki Kim, Yun Kyu Oh, Chun Soo Lim, Yon Su Kim, Sunyoung Kim, Seung Hee Yang, Jung Pyo Lee

**Affiliations:** ^1^ Department of Internal Medicine Hallym University Sacred Heart Hospital Anyang Korea; ^2^ Department of Internal Medicine Seoul National University College of Medicine Seoul Korea; ^3^ Department of Intensive Care Unit Yanbian University Hospital Jilin China; ^4^ R&D Center for Innovative Medicines Helixmith Co., Ltd. Seoul Korea; ^5^ Department of Internal Medicine Seoul National University Boramae Medical Center Seoul Korea; ^6^ Department of Internal Medicine Seoul National University Hospital Seoul Korea; ^7^ Kidney Research Institute Seoul National University Seoul Korea; ^8^ Biomedical Research Institute Seoul National University Hospital Seoul Korea

**Keywords:** acute kidney injury, apoptosis, cMet agonistic antibody, PI3K/Akt/mTOR pathway

## Abstract

Acute kidney injury (AKI) is a very common complication with high morbidity and mortality rates and no fundamental treatment. In this study, we investigated whether the hepatocyte growth factor (HGF)/cMet pathway is associated with the development of AKI and how the administration of a cMet agonistic antibody (Ab) affects an AKI model. In the analysis using human blood samples, cMet and HGF levels were found to be significantly increased in the AKI group, regardless of underlying renal function. The administration of a cMet agonistic Ab improved the functional and histological changes after bilateral ischaemia‐reperfusion injury. TUNEL‐positive cells and Bax/Bcl‐2 ratio were also reduced by cMet agonistic Ab treatment. In addition, cMet agonistic Ab treatment significantly increased the levels of PI3K, Akt and mTOR. Furthermore, after 24 hours of hypoxia induction in human proximal tubular epithelial cells, treatment with the cMet agonistic Ab also showed dose‐dependent antiapoptotic effects similar to those of the recombinant HGF treatment. Even when the HGF axis was blocked with a HGF‐blocking Ab, the cMet agonistic Ab showed an independent dose‐dependent antiapoptotic effect. In conclusion, cMet expression is associated with the occurrence of AKI. cMet agonistic Ab treatment attenuates the severity of AKI through the PI3K/Akt/mTOR pathway and improves apoptosis. cMet agonistic Ab may have important significance for the treatment of AKI.

## 1 INTRODUCTION

The incidence of acute kidney injury (AKI) is increasing every year, regardless of age or sex, and it occurs in 5%‐10% of hospitalized patients and approximately 30% of critically ill patients. Patients with AKI may recover completely but can progress to chronic kidney disease (CKD) or end‐stage renal disease (ESRD) and is an independent risk factor for mortality.^1^

The mechanisms of AKI include tubular injury, endothelial cell activation, tubular obstruction, leucocyte recruitment, vascular injury and the involvement of various immune cells.^2^, ^3^ Thereafter, if tissue repair processes such as macrophage M1 to M2 switching, resolution of inflammatory cell infiltration, tubular proliferation and endothelial repair/regeneration do not occur properly, fibrosis may occur.^4^ Although there have been studies on therapies targeting AKI pathogenesis that prevent AKI from developing and reduce its progression, there is still no effective fundamental treatment.^5^ The elimination of the cause and treatment of the symptoms are the only treatment options.

cMet is a transmembrane tyrosine kinase receptor for hepatocyte growth factor (HGF) and is known to be involved in cell survival, cell growth and regeneration.^6^, ^7^ The HGF/cMet axis is expected to fundamentally improve various causes of kidney disease by inhibiting oxidative stress, apoptosis, fibrosis and inflammation.^8^, ^9^ In particular, cMet monoclonal antibody (Ab), which overcomes the limits of recombinant HGF (rHGF; short half‐life and difficult to purify to biologically stable form),^10^ has been suggested as a potential therapeutic agent.

Recently, authors have confirmed the involvement of the HGF/cMet pathway in kidney fibrosis and verified that the administration of a cMet agonistic Ab reduces fibrosis in primary cultured glomerular endothelial cells (GEnCs)^11^ and proximal tubular epithelial cells (PTECs) and improves fibrosis and apoptosis in a unilateral ureteral obstruction model.^12^ However, there have been no reports on the efficacy of cMet agonistic Ab in an AKI model.

Therefore, in this study, we investigated the involvement of the HGF/Met pathway in AKI and whether treatment with cMet agonistic Ab improves the incidence, severity and course of AKI in a bilateral ischaemia‐reperfusion injury (IRI) model and an in vitro model with human PTECs (hPTECs) and GEnCs. In addition, the mechanism underlying the effect was determined.

## 2 MATERIALS AND METHODS

### 2.1 Measurement of plasma HGF and cMet levels

Among severe AKI patients who started continuous renal replacement therapy, 48 patients with available plasma samples that were collected at the time of AKI diagnosis were enrolled in this study. ESRD patients undergoing permanent dialysis were excluded. Twenty‐four patients with no underlying kidney disease and stable kidney function were enrolled as normal controls. Plasma HGF (catalog no. DY294, R&D Systems) and cMet levels (catalog no. KHO 2031, Thermo Fisher Scientific Inc) were measured using an enzyme‐linked immunosorbent assay according to the manufacturer's instructions. All measurements were performed in a blinded manner in duplicate.

This study was approved by the Institutional Review Board of Seoul National University Boramae Medical Center (no. 20‐2019‐79/112), and all patients provided informed consent prior to enrolment. All clinical investigations were conducted in accordance with the guidelines of the 2013 Declaration of Helsinki.

### 2.2 Animals

Seven‐ to eight‐week‐old male wild‐type mice (C57BL/6; B6) were purchased from Koatech and were raised in a specific pathogen‐free animal facility. All experiments were performed in accordance with the Guidelines for the Care and Use of Laboratory Animals of the National Research Council and the National Institutes of Health under the approval of the Institutional Animal Care and Use Committee of the Clinical Research Institute at Seoul National University Boramae Medical Center (no. 2019‐0003).

An AKI model induced by bilateral IRI was used in this study. The mice were anaesthetized with xylazine (Rompun; 10 mg/kg; Bayer) and Zoletil™ (30 mg/kg; Virbac); then, the kidney pedicles were exposed on both sides through a bilateral flank incision, the pedicles were clipped for 23 minutes using microaneurysm clamps (Roboz Surgical Instrument Co.) and the mice were placed on a heating pad at 38‐39°C in the supine position to maintain body temperature. For optimal fluid balance, pre‐warmed (37°C) PBS (500 µL) was administered intraperitoneally.^13^ Sham‐operated mice received the same surgical procedure except for renal pedicle clamping.

The mice were randomly divided into three groups: (a) sham, which only had a flank incision; (b) bilateral IRI, in which the mice underwent renal ischaemia for 23 minutes; and (c) IRI with cMet agonistic Ab, in which the mice underwent renal ischaemia after cMet agonistic Ab treatment. The cMet agonistic Ab (20 mg/kg) was injected 24 hours and 3 hours before surgery. Blood samples were collected at 24 and 48 hours post‐operatively, and kidneys were also harvested after 48 hours. Serum creatinine (sCr) and blood urea nitrogen (BUN) levels were determined by using an i‐STAT handheld blood analyzer system (Abbot Point of Care Inc) after 24 hours and 48 hours of disease induction, according to the manufacturer's protocol.

### 2.3 Histology

Paraffin‐embedded kidney sections (4‐μm thickness) were stained with a periodic acid‐Schiff (PAS) reagent kit (ScyTek) according to standard protocol. Tubular injury was assessed by a kidney pathologist blinded to the experimental groups using a five‐point scale as follows: score 0, normal; score 1, ≤25%; score 2, 25% < ≤50%; score 3, 50% < ≤75%; and score 4, >75%.

### 2.4 Confocal microscopic examination

Apoptotic cells were detected by TUNEL staining utilizing an ApopTag Fluorescein In Situ Apoptosis Detection kit (Roche). Paraffin‐embedded kidney sections were deparaffinized, hydrated and then gradually incubated with protease K (Dako), terminal deoxynucleotidyl transferase (TdT) enzyme and anti‐digoxigenin‐fluorescein (Roche). Finally, the slides were mounted with Gold antifade reagent containing 4′,6‐diamidino‐2‐phenylindole (DAPI) (Invitrogen) and examined under a fluorescence microscope. For each sample, 5 fields (400×) were randomly selected and quantified using computer‐based morphometric analysis (Qwin 3, Leica). Scoring was performed in a blinded manner using the mean values of the positive areas (%).

The kidney sections were probed with immunofluorescence antibodies against cleaved caspase‐3 (Cell Signaling Technology), CD31 (Novus Biologicals) as a marker of GEnCs, and phospho‐p21 (Santa Cruz Biotechnology) in a blocking reagent overnight at 4°C. A second layer of Alexa Fluor® 488‐conjugated goat anti‐rabbit antibody, Alexa Fluor® 555‐conjugated goat anti‐rat antibody and Alexa Fluor® 647‐conjugated goat antimouse antibody (Molecular Probes) was applied, respectively. After 1 hour at room temperature, all sections were washed and then incubated for an additional 5 minutes with DAPI (Sigma‐Aldrich) for counterstaining. For the negative controls, the primary antibodies were omitted from the sections. The sections were evaluated in a blind and random manner, and images were captured using a Leica TCS SP8 STED CW instrument (20×/0.7 NA objective lens of the DMI 6000 inverted microscope; Leica) and MetaMorph version 7.8.10 software (Universal Imaging).

### 2.5 Quantitative real‐time PCR

Total RNA was isolated from hPTECs and kidney tissue using TRIzol reagent (Bioline). cDNA synthesis was performed using a Convenient System for Reverse Transcription kit (Promega) and a C1000 thermal cycler. Subsequently, we performed quantitative real‐time PCR (qRT‐PCR) using a LightCycler‐480 instrument II (Roche Molecular Systems Inc). The PCR probes used in this study were as follows: Bax, Mm.PT.58.5345963; Bcl‐2, Mm.PT.58.7362966; and GAPDH, Mm.PT.58.39a.1. The mRNA levels were calculated using the comparative Ct method (∆∆Ct) after normalization to GAPDH.

### 2.6 Western blotting

Protein samples were extracted from the homogenized kidneys and cells using RIPA buffer (Biosesang) and prepared as equal concentration lysates for electrophoretic separation using the BCA assay was to measure the concentration. The prepared protein samples were electrophoresed with glycine‐SDS buffer (LPS solution) and transferred to PVDF membranes (Millipore Corporation) on ice. After the membranes were blocked with 5% skim milk (Becton Dickinson Rowa France) containing 2% BSA buffer, they were incubated with primary antibodies overnight at 4°C with shaking; E‐cadherin (Abcam), IL‐1β, p‐P38, P38, PI3K, Akt, mTOR and GAPDH (Cell Signaling Technology). The next day, the membranes were incubated with a mouse or rabbit IgG‐conjugated secondary antibody (Cell Signaling Technology) for 1 hour. Finally, the protein bands were observed by the ECL chemiluminescence method (Advansta). Densitometry was performed using the gel analysis procedure in ImageJ (National Institutes of Health).

### 2.7 Cell culture

According to the guidelines approved by the Institutional Review Board of Seoul National University Hospital (no. 1404‐117‐515), hPTECs^3^ and GEnCs^11^ were isolated from normal tissue specimens of resected kidneys from renal cell carcinoma patients. After the cortex was dissected, the tissues were minced and digested in Hank's balanced salt solution (HBSS) containing 3 mg/mL collagenase (Sigma) at 37°C for 1 hour.

Cortical tubular cells were isolated after washing through a series of sieves (150, 120, 70 and 40 μm) with PBS and centrifugation for 5 minutes at 500 *g* and then incubated in DMEM/F12 (Lonza). After 4 hours, the collected tubular epithelial cells were cultured on collagen‐coated Petri dishes (BD Biosciences) until colonies were established, and cells grown for 2‐3 passages were used in this study. After 3 days, the cells were removed from the dishes by treatment with 3 mmol/L EDTA solution and a minimal amount of trypsin and seeded (2 × 10^5^ cells/well) on 6‐well chamber slides. After 24 hours of serum starvation, the cells were washed twice with PBS. Then, 10 ng/mL rHGF (R&D Systems) and a cMet agonistic Ab were used to treat cells at concentrations of 250, 500 and 1000 ng/mL to confirm the expression levels of p‐cMet and cMet.

For GEnCs, minced cortex was serially passed through mesh stainless steel screens (160, 120 and 100 μm), and glomeruli were collected using a 75‐μm mesh screen. Then, glomeruli were suspended in HBSS (Gibco‐BRL Life Technologies), washed twice, centrifuged (800 *g*, 1 min) and digested in 1.5 mg/mL collagenase (Sigma) for 40 minutes at 37°C. Single‐cell suspension of GEnCs was resuspended in RPMI‐1640 (Sigma) supplemented with 20% foetal bovine serum (Gibco‐BRL), 10 ng/mL human basic fibroblast growth factor (Sigma), 10 ng/mL epidermal growth factor (Sigma), 5 ng/mL vascular endothelial growth factor (Peprotech), 1 mg/mL hydrocortisone (Sigma), 20 U/mL heparin, 50 U/mL penicillin and 50 mg/mL streptomycin (Gibco‐BRL) and plated on fibronectin‐coated 35‐mm culture dishes (BD Biosciences). After 1 week, trypsinization was performed until a culture with 98.1% GEnCs was achieved. Then, the cells were maintained in collagen‐coated 100‐mm culture dishes (Iwaki).

### 2.8 Cell cycle analysis

First, cells were treated with rHGF (10 ng/mL) or the cMet agonistic Ab (500 and 1000 ng/mL) and then incubated under hypoxic conditions for 24 hours. Cells cultured under normoxic conditions were used as a negative control, and cells cultured under hypoxic conditions alone were used as a positive control. Second, to check whether the cMet agonistic Ab works without going through the HGF/cMet pathway, an HGF‐blocking Ab (1 μg/mL) was used, and the reaction was confirmed by simultaneously treating the cells with a cMet agonistic Ab. In this case, a group treated with the same amount of IgG (1 μg/mL, R&D Systems) was used as a positive control. After 24 hours, the cells were harvested with 2.21 mmol/L EDTA and 0.25% trypsin, fixed with BD Cytofix/Cytoperm (BD Biosciences) and then stained with DAPI (1 μg/mL). The cell cycle was measured by flow cytometry using a 4‐laser LSRFortessa (BD Biosciences) and analysed by FlowJo software (BD Biosciences).

### 2.9 Annexin V/propidium iodide staining assay

We measured cell apoptosis and necrosis using an Annexin V/propidium iodide (PI) fluorescein isothiocyanate (FITC) apoptosis kit (BD Biosciences) by flow cytometry. As positive controls, human IgG (0.5 μg/mL, R&D Systems) and goat IgG (0.5 μg/mL, R&D Systems) were used. cMet blocking Ab (0.5 μg/mL, R&D Systems) was also used. Harvested hPTECs and GEnCs (5 × 10^5^) were washed with cold PBS, resuspended in 100 μL of binding buffer, stained with 5 μL of FITC‐conjugated Annexin V (10 mg/mL) and 10 μL of PI (50 mg/mL), and incubated for 30 minutes at room temperature in the dark. Then, acquired data were analysed with BD FACSDiva™ (V8.0).

### 2.10 Statistical analysis

The values are expressed as the mean ± standard error of the mean (SEM) or the median (interquartile range) based on the results of the Shapiro‐Wilk normality test. Student's *t* test or the Mann‐Whitney *U* test was also used. Statistical analyses were performed using SPSS version 22 (IBM software, USA) and GraphPad Prism 5.0 (GraphPad Software, Inc, San Diego, CA, USA). Statistical significance was determined at *P < *.05.

## 3 RESULTS

### 3.1 Plasma cMet and HGF levels are associated with the occurrence of AKI

**FIGURE 1:**
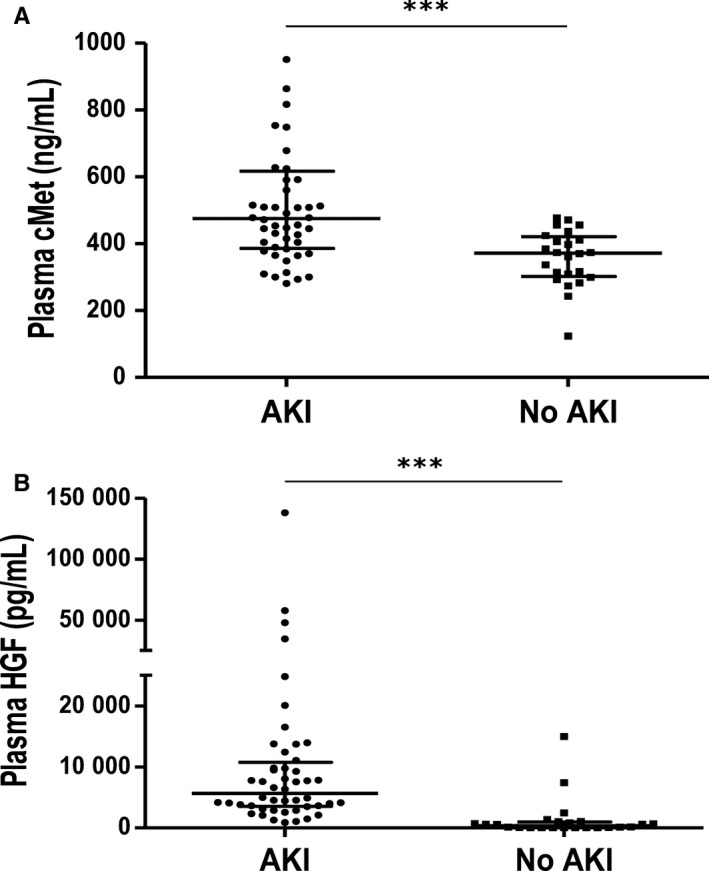
Plasma cMet levels are associated with the occurrence of AKI. (A) The cMet level was measured in forty‐eight AKI and twenty‐four control patient plasma samples. The patients diagnosed with AKI had higher plasma cMet levels than those in the control group (***P < .001). (B) Plasma HGF concentrations were also different between the two groups (***P < .001). All measurements were performed in a blinded manner in duplicate. The results are expressed as the median (interquartile ranges) and were compared with the Mann‐Whitney *U* test

Plasma cMet and HGF levels were measured in 48 AKI patients and 24 patients without AKI (Figure 1). The baseline characteristics of these patients are described in Table 1. There was no statistically significant difference in the underlying renal function or underlying disease between the two groups. At the time of plasma sample collection, which showed a difference in renal function, cMet and HGF levels were significantly increased in the AKI group (*P* < .001). The elevated plasma cMet and HGF levels were associated with the AKI episode.

**TABLE 1:** Baseline characteristics

	Acute kidney injury (n = 48)	No acute kidney injury (n = 24)	*P*
Male sex	29 (60.4)	17 (70.8)	.386
Age (y)	61 (48, 74)	71 (57, 76)	.203
Body mass index (kg/m^2^)	23.3 (20.8, 27.1)	24.1 (22.0, 27.0)	.591
Baseline sCr (mg/dL)	0.98 (0.71, 1.50)	0.83 (0.73, 0.93)	.057
Baseline MDRD‐GFR (mL/min/1.73 m^2^)	72.7 (43.0, 105.2)	85.5 (77.7, 91.2)	.068
Chronic kidney disease			.030
Stage 1	12 (25.0)	8 (33.3)	
Stage 2	13 (27.1)	16 (66.7)	
Stage 3	12 (25.0)	0 (0.0)	
Stage 4	3 (6.2)	0 (0.0)	
Unknown	8 (16.7)	0 (0.0)	
Hypertension	25 (52.1)	15 (62.5)	.402
Diabetes mellitus	21 (43.8)	7 (29.2)	.231
Liver disease	10 (20.8)	1 (4.2)	.064
Systolic BP (mm Hg)	107 (92, 125)	139 (117, 166)	<.001
Serum creatinine (mg/dL)	2.65 (1.82, 3.83)	0.99 (0.84, 1.12)	<.001
MDRD‐GFR (mL/min/1.73 m^2^)	21.5 (15.2, 36.0)	73.7 (56.9, 84.4)	<.001
Plasma cMet (ng/mL)	475.0 (385.9, 616.7)	371.9 (302.3, 421.3)	<.001
Plasma HGF (pg/mL)	5659.2 (3533.1, 10 757.7)	353.4 (32.8, 958.6)	<.001

Note

The data are presented as the median (25th and 75th percentiles) or as a number (percentage, %).

Abbreviations: BP, blood pressure; GFR, glomerular filtration rate; HGF, hepatocyte growth factor; sCr, serum creatinine.

### 3.2 cMet agonistic Ab alleviates IRI‐induced renal function deterioration

**FIGURE 2:**
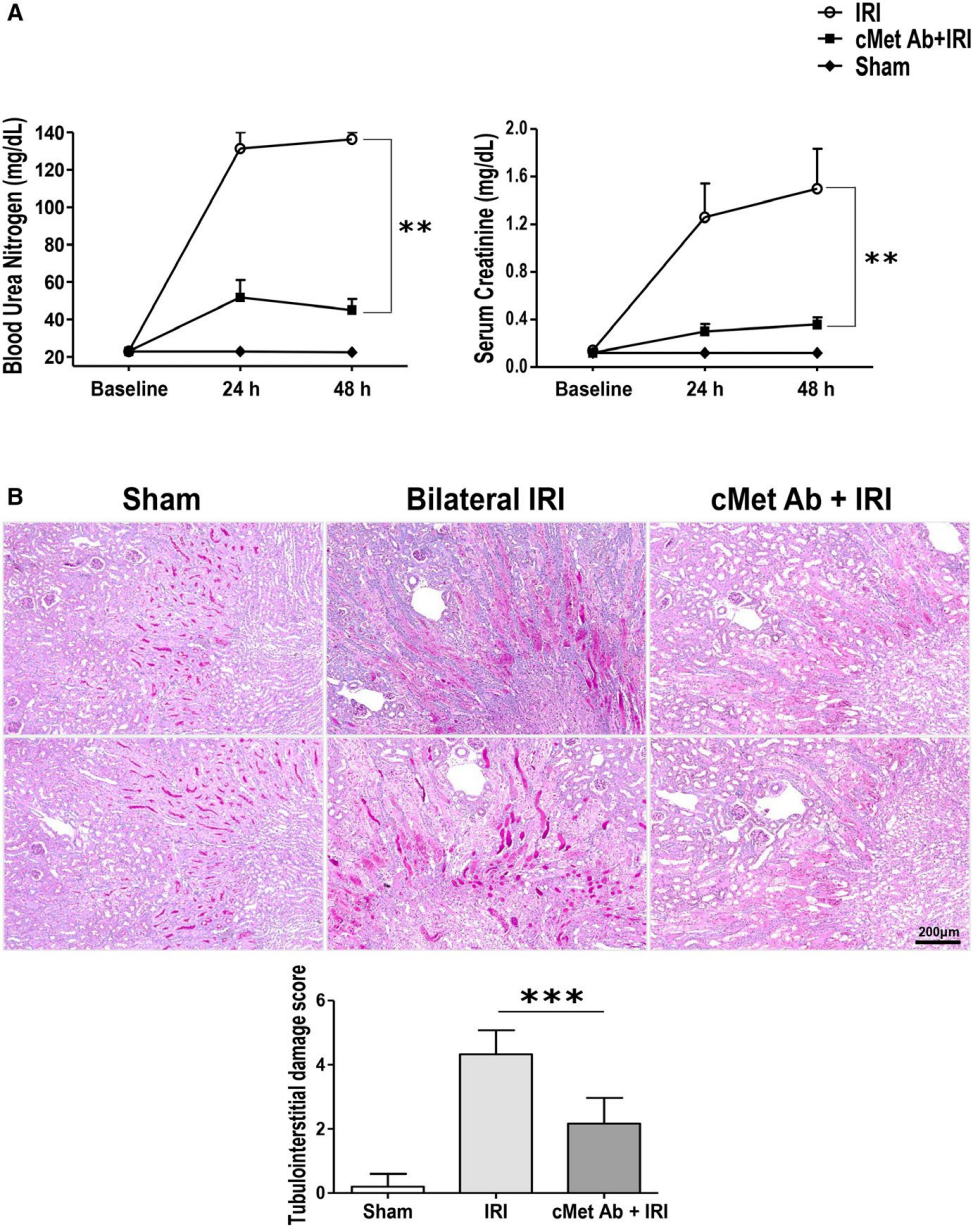
cMet agonistic Ab treatment attenuates the severity of AKI. (A) Functional data. After bilateral IRI induction, BUN and sCr levels steadily increased and peaked at 48 h. However, the values were significantly lower in the cMet agonistic antibody‐injected group than the IRI group (**P < .01; n = 10/group). (B) PAS stain. After 48 h of disease induction, tubular necrosis and inflammation were less extensive in the cMet agonistic antibody‐injected group than the bilateral IRI group (n = 10/group; ***P < .001; magnification: 100×). The data are shown as the mean ± SEM and were compared using Student's *t* test

The IRI group showed significantly higher levels of sCr and BUN than the control groups at 24 hours and 48 hours after surgery. However, in the IRI mice treated with a cMet agonistic Ab, the sCr and BUN levels were significantly lower than those in the IRI only mice (Figure 2A).

Similar to the functional data, the PAS staining showed structural disruptions such as brush border loss, cast formation in tubules and infiltration of inflammatory cells in the IRI group (Figure 2B). In contrast, cMet agonistic Ab treatment improved the kidney morphology changes. These changes were all demonstrated by histological scoring.

### 3.3 cMet agonistic Ab improves apoptosis induced by IRI

**FIGURE 3:**
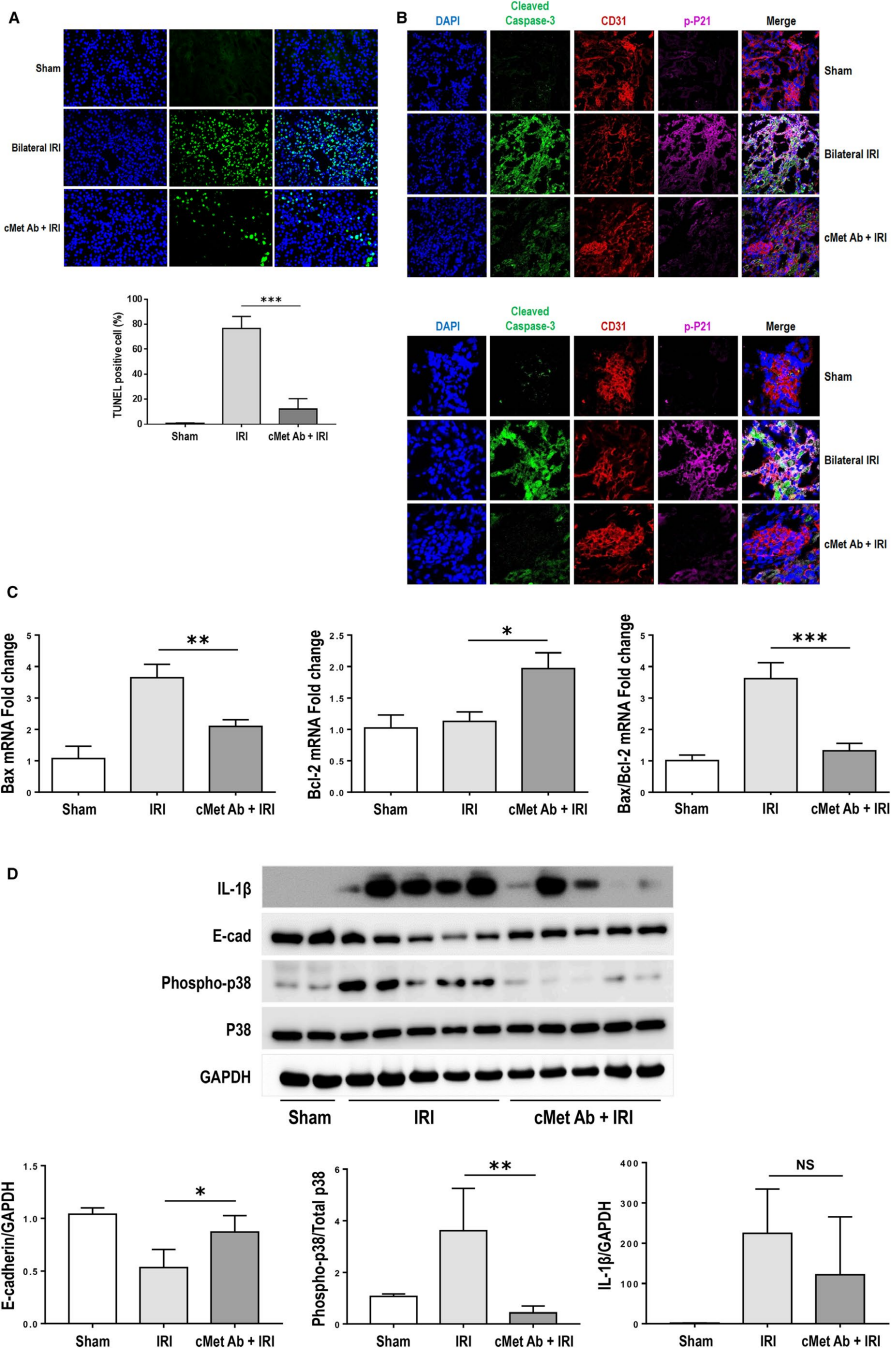
cMet agonistic Ab treatment improves apoptosis in AKI. (A) TUNEL assay. The percentage of TUNEL‐positive cells was significantly decreased in the cMet agonistic Ab‐injected group compared with the bilateral IRI group (n = 10/group; ***P < .001; magnification: 400×). (B) Apoptosis in GEnCs. The IRI group showed the decrease in CD31 expression and the increase in the expression of cleaved caspase‐3 and p‐P21. However, in the mice treated with cMet agonistic Ab, CD31 expression was enhanced and the expression of cleaved caspase‐3 or p‐P21 was decreased (magnification: 400×, 1000×). (C) Bax/Bcl‐2 ratio. The Bax/Bcl‐2 ratio was increased in the IRI group and markedly decreased in the cMet agonistic Ab‐treated group (n = 10/group; *P < .05, **P < .01, ***P < .001). (D) The band of representative samples and quantification of results showed that IL‐1β, p‐P38 and P38 expression was elevated in the IRI group compared with the control group and prominently attenuated by pre‐treatment with the cMet agonistic Ab. In contrast, E‐cadherin expression showed the opposite result. All data are shown as the mean ± SEM and compared using Student's *t* test (n = 10/group; *P < .05, **P < .01). The results shown are one of three independent experiments with the same trend

TUNEL staining, which reveals DNA fragmentation in apoptosis, showed that the percentage of apoptotic cells was higher in the IRI group than the sham group and markedly decreased after treatment with the cMet agonistic Ab (Figure 3A). As shown in Figure 3B, CD31 expression was decreased and expression of cleaved caspase‐3 and p‐P21 was increased as a result of apoptosis of GEnCs in the IRI group than the sham group. In contrast, the GEnCs from the mice treated with cMet agonistic Ab stained more intensely and exhibited decreased expression of cleaved caspase‐3 or p‐P21.

The Bax/Bcl‐2 ratio, which determines cell susceptibility to apoptosis, was examined by qRT‐PCR (Figure 3C). The Bax/Bcl‐2 ratio was increased in the IRI group and significantly decreased in the cMet agonistic Ab‐treated group, similar to the results of the sham group. In addition, cMet agonistic Ab treatment induced a decrease in the p‐P38 and IL‐1β levels, accompanied by an increase in the E‐cadherin level (Figure 3D).

### 3.4 cMet agonistic Ab increases PI3K/Akt expression on IRI‐induced kidney injury

**FIGURE 4:**
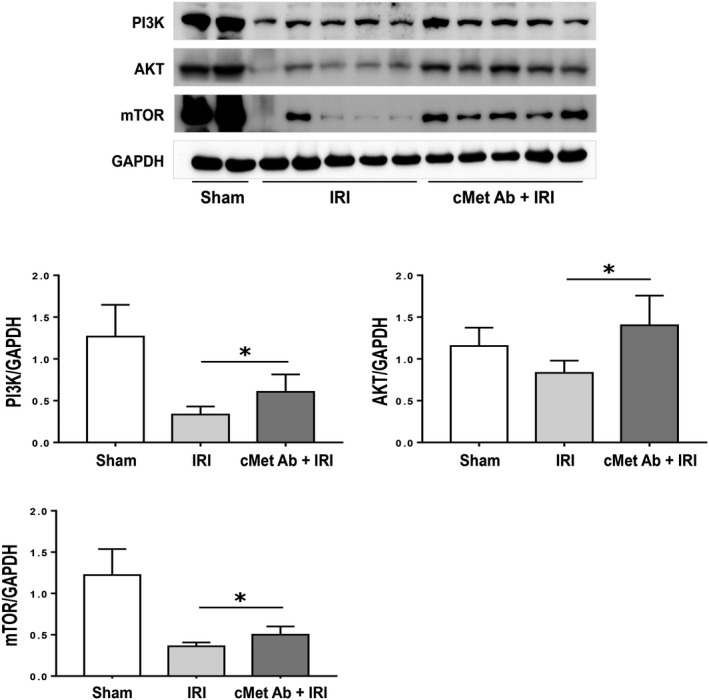
cMet agonistic Ab treatment activates the PI3K/Akt pathway. The IRI group showed significantly decreased levels of PI3K, Akt and mTOR compared to the sham group. In contrast, the levels of these proteins were markedly increased by cMet agonistic Ab treatment. All data are shown as the mean ± SEM and compared using Student's *t* test (n = 10/group; *P < .05). The results shown are one of three independent experiments with the same trend

Next, we measured the levels of Akt, PI3K and mTOR to explore whether the antiapoptotic effects of the cMet agonistic Ab are associated with the PI3K/Akt/mTOR pathway. The IRI group showed significantly lower protein levels of PI3K, Akt and mTOR than the sham group; however, the levels of these proteins increased significantly after cMet agonistic Ab treatment (Figure 4).

### 3.5 *Effects of the cMet agonistic Ab on apoptosis in *in vitro* study*

**FIGURE 5:**
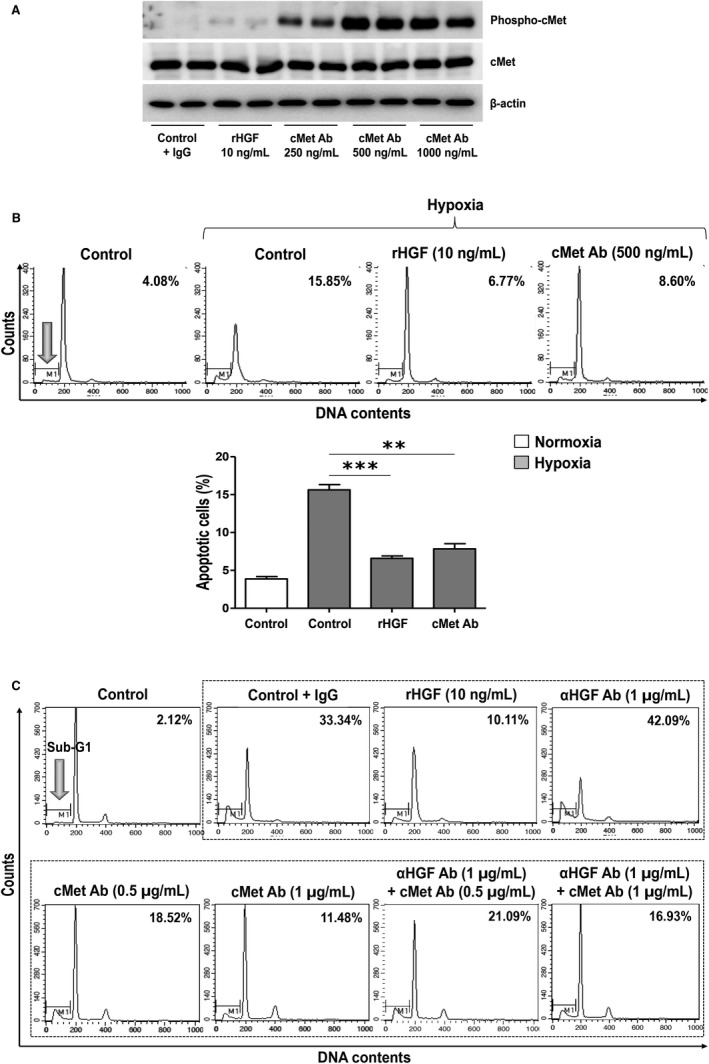
Effects of cMet agonistic Ab treatment on apoptosis in in vitro study. (A) The treatment of hPTECs with the cMet agonistic Ab increased the level of p‐cMet, suggesting that this antibody is functional in hPTECs. (B) Antiapoptotic effect of cMet agonistic Ab treatment. In hypoxia‐injured hPTECs, the apoptosis rate was higher than that in the control hPTECs. Treatment with the cMet agonistic Ab again reduced the proportion of apoptotic cells to a level comparable with that of rHGF treatment (**P < .01, ***P < .001). (C) cMet agonistic Ab is also effective when cells are treated with an HGF‐blocking antibody. After hypoxic stimulation and treatment with the cMet agonistic Ab, the percentage of apoptotic cells decreased dose‐dependently to a level comparable with that of rHGF treatment. The increase in the proportion of apoptotic cells after treatment with an HGF‐blocking antibody significantly decreased after cMet agonistic antibody treatment. These results are representative of one of three independent experiments

Treatment with the cMet agonistic Ab dose‐dependently increased p‐cMet, which was significantly different from that produced by treatment with recombinant HGF (Figure 5A). In hPTECs cultured under hypoxic conditions, 15.85% of the cells were apoptotic, which was approximately four  times higher than in the control conditions, and the percentage of apoptotic cells decreased by half after treatment with the cMet agonistic Ab. This result was similar to that of rHGF treatment which reduced the proportion of apoptotic cells to 6.77% (Figure 5B).

Next, cells were simultaneously treated with an HGF‐blocking Ab to exclude the effect of HGF that is re‐secreted from cells (Figure 5C). After 24 hours of hypoxia induction in PTECs, cMet agonistic Ab treatment also showed dose‐dependent antiapoptotic effects, which were similar to those of the rHGF treatment; however, the antiapoptotic effect was attenuated by the HGF‐blocking Ab treatment. Even when the HGF axis was blocked with a HGF‐blocking Ab, the cMet agonistic Ab produced an independent dose‐dependent antiapoptotic effect.

**FIGURE 6:**
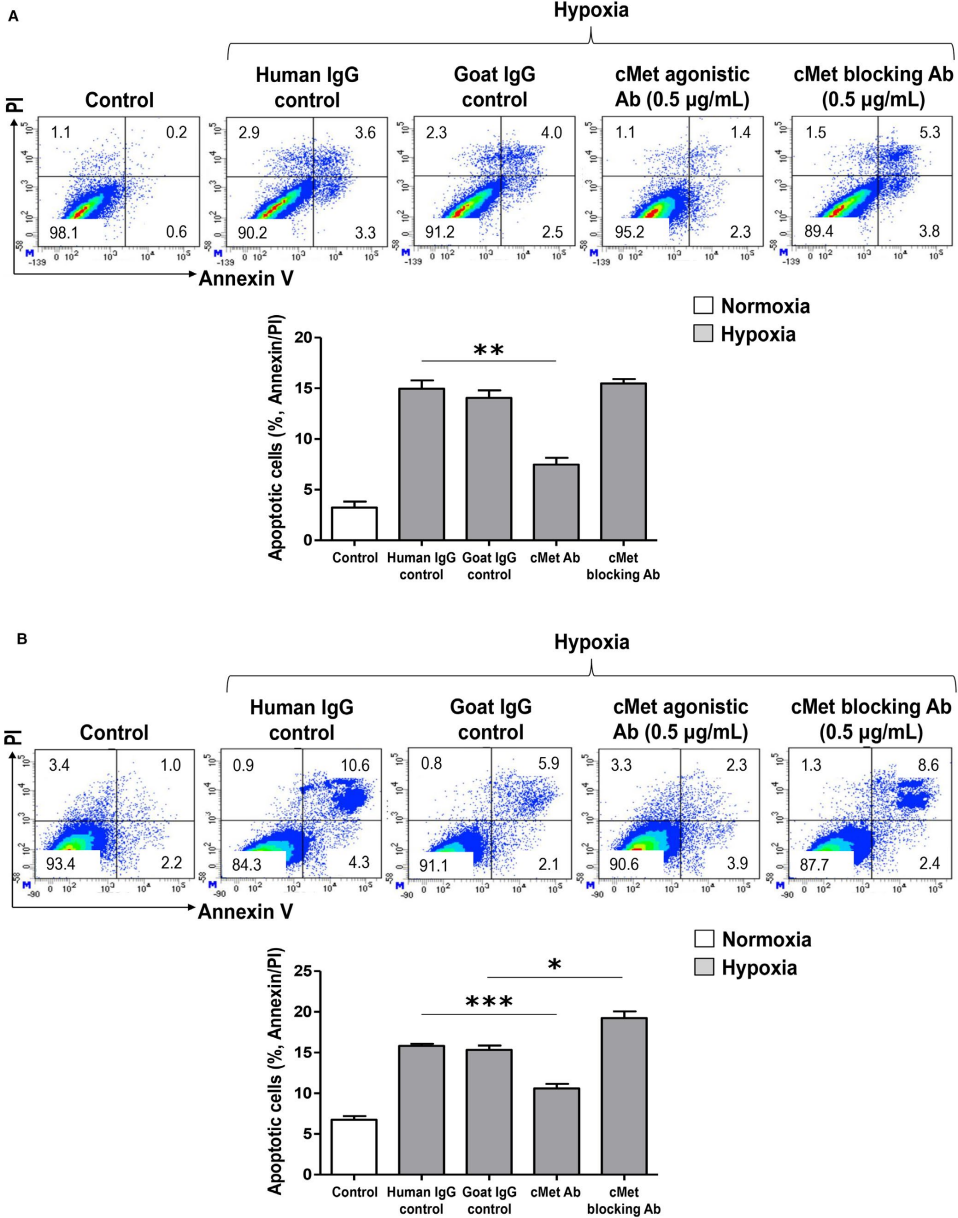
Annexin V/propidium iodide staining assay. (A) In the hypoxic condition of hPTECs, apoptotic cells decreased under the cMet agonistic Ab treatment compared with those under human IgG control treatment; however, when treated with cMet blocking Ab, apoptotic cells showed an increased pattern compared to the Goat IgG control (**P < .01). (B) Treatment of cMet agonistic Ab to GEnCs also significantly reduced apoptotic cells; in contrast, the increased apoptotic cells under hypoxia significantly decreased following the treatment of the cMet blocking Ab (*P < .05, ***P < .001). These results are representative of one of three independent experiments

The antiapoptotic effect of the cMet agonistic Ab was also demonstrated by Annexin V/propidium iodide staining assay using hPTECs (Figure 6A) and GEnCs (Figure 6B). On the other hand, when treated with cMet blocking Ab it was confirmed that the apoptotic cells tend to increase significantly more than the control IgG treatment.

## 4 DISCUSSION

In this study, we confirmed that cMet and HGF expression increases when AKI occurs. Treatment with a cMet agonistic Ab produced a decrease in the severity of AKI, improved histological changes and improved the inflammatory response, especially apoptosis. We also found that the PI3K/Akt/mTOR pathway is involved. Even when HGF activity was blocked, cMet agonistic Ab treatment produced the same effect, suggesting that the cMet agonistic Ab functions independently of HGF.

Acute kidney injury is a common kidney disease with many clinical side effects, but there is no fundamental treatment, so long‐term effects occur; therefore, the resolution of this disease is very important in terms of nephrology, and there has been much research to identify its mechanism.^14^, ^15^, ^16^

Soluble cMet has been studied for its role in many diseases. The levels of cMet, which interacts with several proteins that promote cell migration, in urine samples from patients with metastatic prostate cancer have been shown to be very high, suggesting that this may be a biomarker and a major regulator of cancer progression.^17^ However, Zeng et al^18^ reported that the soluble cMet level is as an early protective biomarker in patients with severe pre‐eclampsia, suggesting that cMet is expressed as a kind of feedback signal to protect endothelial cells after injury. Recently, Yang et al^19^ reported that in a Korean multicenter cancer cohort, the absolute risk of gastric cancer was reduced in patients with high levels of soluble cMet protein. The soluble cMet level is significantly higher in healthy controls than cancer patients and gradually decreases over time after cancer diagnosis, suggesting the antitumour potential of soluble cMet protein. We also recently reported that the urinary cMet levels measured at initial diagnosis in diabetic nephropathy predict renal outcome.^11^

In this study, soluble cMet levels were found to be increased in patients with AKI but not in patients without AKI. The increase in cMet in patients with AKI may be interpreted as a protection and recovery marker after injury rather than simply as an injury marker. The similarly increased HGF level supports this conclusion. To investigate this hypothesis, an in vivo AKI model was established in mice, which were treated with a cMet agonistic Ab to increase cMet activity.

It has been shown in traditional studies that HGF promotes recovery in AKI animal models.^20^ In addition, in the drug‐induced AKI model, HGF administration improves the kidney damage and increases the survival rate, but the short half‐life of HGF produces the limitation that frequent administration is required.^21^ In experiments with HUVECs, Gong et al^22^ reported that pre‐treatment with HGF reduces acute inflammatory responses. Recently, bilateral IRI and cisplatin‐induced AKI in cMet knockout mice have been shown to produce more severe kidney damage and worsen apoptosis or inflammatory response. This suggests that the activation of the HGF receptor cMet in the renal tubules has a protective effect on the kidneys.^23^

In our study, the extent of kidney damage was reduced, and apoptosis and inflammatory responses were also restored by the activation of the cMet pathway with the cMet agonistic Ab. Previous studies have shown that apoptosis plays a crucial role in the development of hypoxia‐reoxygenation injury in renal tubular epithelial cells.^24^ Pro‐apoptotic Bax is essential for the cell death progress, and cell viability depends to a large extent on the interactions between Bcl‐2 family proteins, as well as the sensitivity of cells to apoptosis.^25^ We found that treatment with the cMet agonistic Ab decreased the Bax/Bcl‐2 ratio and percentage of TUNEL‐positive cells with apoptotic DNA fragmentation. These results suggest that the cMet agonistic Ab can markedly ameliorate apoptosis. The effect of cMet agonistic Ab observed in both hPTECs and GEnCs, was cancelled by cMet blocking Ab, rather increased apoptosis.

Moreover, many studies have shown that inhibiting the cell death mechanism induced by the activation of the PI3K/Akt pathway protects against IRI.^26^, ^27^, ^28^, ^29^ PI3K/Akt/mTOR phosphorylation usually promotes survival through the activation of antiapoptotic proteins and the inhibition of pro‐apoptotic proteins in AKI model.^26^ We also found that cMet agonistic Ab treatment can protect against IRI‐induced kidney damage by activating the PI3K/Akt/mTOR pathway, which affects apoptosis. Notably, although we blocked HGF in the experiment using hPTECs, the cMet Ab effects were retained, which may be important evidence that the cMet agonistic Ab can work independently of HGF.

We did not elucidate a new mechanism of AKI nor establish a new AKI model but did demonstrate the protective effect of the administration of a cMet agonistic Ab against kidney damage in an AKI model induced by bilateral IRI. Furthermore, overcoming the short half‐life limitations of HGF treatment and seeing injury improvement with only two Ab doses and without gene therapy such as gene knock out, is encouraging. Although the antibody dose is high and the dose‐dependent effect has not been confirmed in animal experiments, cMet agonistic Ab with the highest binding activity to cMet has been selected from antibody production process and used at the doses established in previous studies. The possibility of cMet agonistic Ab side effects, such as inflammation, cell proliferation and angiogenesis, has already been confirmed in other experiments.^12^ Finally, if the experiments were performed using conditional knockout mice of the cMet gene, this hypothesis could be further confirmed.

In conclusion, the expression of cMet is closely linked to AKI development. cMet agonistic Ab treatment attenuated the severity of AKI through the PI3K/Akt/mTOR pathway and ameliorated the inflammation and apoptosis associated with AKI. cMet and cMet agonistic Ab may have important significance for the severity assessment and treatment of AKI.

## CONFLICT OF INTEREST

The authors declare no competing interests.

## AUTHOR CONTRIBUTIONS

JNA, LL, SHY and JPL conceived the study. JPL acquired funding. JL, SY and SK provided resources. JNA, LL, JL, YCK, SHY and JPL involved in investigation and methodology. JNA and SHY involved in formal analysis and visualization. JL, SY, DKK, YKO, CSL, YSK and SK supervised and validated the study. JNA, LL, SHY and JPL wrote the manuscript. All the authors listed have reviewed the final version of the manuscript and approved it for submission.

## Data Availability

All other data supporting the presented findings are available from the corresponding author upon request.
